# Similar efficacy and safety between lenvatinib versus atezolizumab plus bevacizumab as the first‐line treatment for unresectable hepatocellular carcinoma

**DOI:** 10.1002/cam4.5506

**Published:** 2022-12-05

**Authors:** Chung‐Wei Su, Wei Teng, Po‐Ting Lin, Wen‐Juei Jeng, Kuei‐An Chen, Yi‐Chung Hsieh, Wei‐Ting Chen, Ming‐Mo Ho, Chia‐Hsun Hsieh, Ching‐Ting Wang, Pei‐Mei Chai, Chen‐Chun Lin, Chun‐Yen Lin, Shi‐Ming Lin

**Affiliations:** ^1^ Department of Gastroenterology and Hepatology Chang Gung Memorial Hospital, Linkou Medical Center Taoyuan Taiwan; ^2^ College of Medicine, Chang Gung University Taoyuan Taiwan; ^3^ Department of Medical Imaging and Intervention Chang Gung Memorial Hospital, Linkou Medical Center Taoyuan Taiwan; ^4^ Department of Medical Oncology Chang Gung Memorial Hospital, Linkou Medical Center Taoyuan Taiwan; ^5^ Department of Medical Oncology Tucheng Composite Municipal Hospital New Taipei Taiwan; ^6^ Department of Nursing Chang Gung Memorial Hospital, Linkou Medical Center Taoyuan Taiwan

**Keywords:** adverse event, atezolizumab plus bevacizumab, hepatocellular carcinoma, lenvatinib, survival

## Abstract

**Background:**

Lenvatinib and atezolizumab plus bevacizumab(A + B) have been used for unresectable hepatocellular carcinoma (HCC) as first‐line therapy. Real‐world studies comparison of efficacy and safety in these two regimens are limited, we therefore conduct this study to investigate these issues.

**Methods:**

We retrospectively reviewed patients received lenvatinib (*n* = 46) and A + B (*n* = 46) as first‐line systemic therapy for unresectable HCC in a tertiary medical center. Objective response rate (ORR), progression free survival (PFS), and overall survival (OS) were evaluated according to modified Response Evaluation Criteria in Solid Tumors (mRECIST). Inverse probability weighting (IPW) was performed for baseline clinical features balance.

**Results:**

A total of 92 patients with median age of 63.8 year‐old, 78.3% male, 85.9% viral hepatitis infected, 67.4% BCLC stage C were enrolled. The median treatment and follow‐up duration were 4.7 months and 9.4 months, respectively. There was no significant difference in ORR (26.1% vs. 41.3%, *p* = 0.1226), PFS (5.9 vs. 5.3 months, *p* = 0.4066), and OS (not reached vs. not reached, *p* = 0.7128) between the lenvatinib and A + B groups. After IPW, the results of survival and response rate were also compared. Subgroup analysis suggested that using lenvatinib was not inferior to A + B in regards of PFS, including those with elder, Child‐Pugh class B, beyond up‐to‐seven, or portal vein invasion VP4 patients. Among the lenvatinib treated patients, multivariate analysis showed patients elder than 65‐year‐old was an independent predictor associated with shorter PFS (adjust HR: 2.085[0.914–4.753], *p* = 0.0213). The incidence rates of adverse events were similar between two groups (76 vs. 63%, *p* = 0.1740). Both of two regimens had similarly few impact on liver function by comparison of baseline, third month, and sixth month albumin‐bilirubin index and Child‐Pugh score.

**Conclusions:**

The efficacy and safety of lenvatinib are similar to A + B as a first‐line systemic therapy for unresectable HCC.

## Graphical abstract

This study provides real‐world experience of lenvatinib and A + B as firstline treatment for unresectable HCC. The data showed that compared survival, response rate, and adverse events between two regimens.

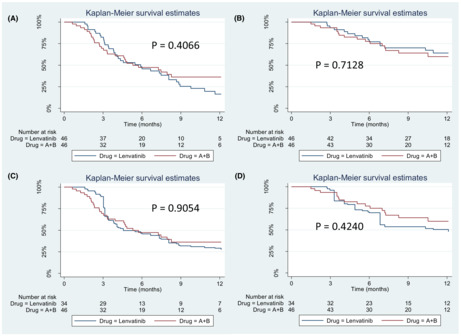

## 1 INTRODUCTION

Hepatocellular carcinoma (HCC) has been the major adverse liver event among patients with chronic viral hepatitis infection for its being the fourth leading cause of cancer‐related death.^1^ HCC could be cured by tumor resection, ablation, or liver transplantation in early stage. On the contrary, appropriately treatment for advanced HCC patients is still a troublesome issue due to poor prognosis. Sorafenib, a muti‐kinase inhibitor (MKI) which can target several protein receptors to interfere tumor proliferation was widely used to treat advanced HCC since approved in 2007 SHARP trial.^2^ A randomized control phase III trial, REFLECT provided another choice of MKI, lenvatinib with a successfully noninferior overall survival (OS) outcome as compared to sorafenib in 2018 for first‐line treatment of advanced HCC (median OS 13.6 months versus 12.3 months).^3^ Significantly improved median progression survival (PFS), time to progression (TTP), and increased objective response rate (ORR) all indicating lenvatinib is a potentially highly effective first‐line therapy for advanced HCC.

Recently, studies of immune checkpoint inhibitor (ICI) for advanced HCC were emerging, offering different strategies of tumor clearance by boosting the immune system. As monotherapy of ICI, nivolumab and pembrolizumab are both belonged to the programmed death receptor‐1 (PD1) class of inhibitors providing 15%–18% ORR and prolonging median OS to 14–16 months.^4^, ^5^ For better response and survival benefit, combination strategies gained more attention in these years. As an example, antiangiogenic agents might enhance the efficacy of ICI though vessel normalization and ICI sensitize tumors to antiangiogenic therapy.^6^ A recent study, IMbrave150, combination therapy with atezolizumab (a PDL1 inhibitor) plus bevacizumab (a vascular endothelial growth factor inhibitor, anti‐VEGF) increasing ORR up to 33% with 6.8 months of median PFS, resulted in better survival advantage than sorafenib alone.^7^ The therapeutic efficacy was also confirmed by several real‐world studies including our groups.^8^, ^9^, ^10^ Since current treatment recommendation suggested A + B as first choice and lenvatinib was alternative choice, which systemic therapy would be the first regimen of choice for advanced HCC patients remained unclear due to lack of head‐to‐head comparison.^11^ This study aimed to compare the therapeutic efficacy and safety between patients with advanced HCC treated by lenvatinib and A + B as first‐line systemic treatment.

## 2 MATERIALS AND METHODS

### 2.1 Patient recruitment

**FIGURE 1:**
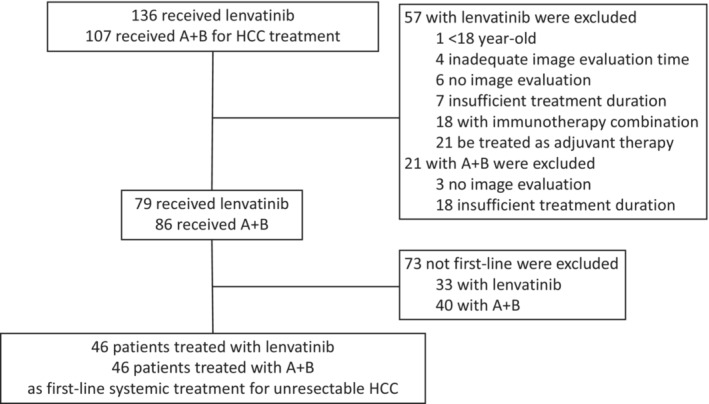
Patient recruitment of the study. A + B, atezolizumab plus bevacizumab; HCC, hepatocellular carcinoma.

We retrospectively reviewed the medical records of intermediate to advanced stage HCC patients who received lenvatinib (*N* = 136) and A + B (*N* = 107) for HCC between May 2018 and March 2022 at a tertiary medical center in Taiwan. The diagnosis of HCC as well as its staging were based on pathology or image criteria of the American Association for the Study of Liver Disease,^12^ belonged to Barcelona clinic liver cancer (BCLC) stage C or not amenable to locoregional therapy in BCLC stage B such as those patients with impaired renal function (Creatinine ≥2 mg/dL or creatinine clearance <30 ml/min), insufficient response after ≥2 consecutive transarterial chemoembolization (TACE) or diffuse, infiltrative HCC with liver involvement. Patients who received less than a month of lenvatinib or only one course of A + B due to impaired liver function as Child‐Pugh class C or deteriorated performance status as the Eastern Cooperative Oncology Group (ECOG) of 3, as an adjuvant therapy after curative ablation, < 18 year old, combination with lenvatinib with immunotherapy, inadequate image evaluation time, no image evaluation, and underwent systemic treatment previously were excluded. Finally, 46 patients received lenvatinib and 46 patients received A + B as first‐line systemic treatment for advanced HCC were included in the analysis (Figure 1). HCC treatment suggestions were discussed in detail in our multidisciplinary meetings with the hepatologist, surgeon, medical oncologist, radiational oncologist, radiologist, and pathologist, before the shared decision making by patients and doctors discussion. The study has been approved by the institutional review board of Chang Gung Medical Foundation approved the study.

### 2.2 Treatment and follow‐up protocol

The daily dosage of lenvatinib was initially prescribed orally 12 mg (body weight ≥ 60 kg) or 8 mg (body weight ≤ 60 kg) as standard with modified based on liver function and the experience of clinical physician, and then was adjusted by patients' tolerance. Initial 8‐week relative dose intensity (8 W‐RDI) was defined as the ratio of the actual dose delivered during the initial 8 weeks to the standard dose.^13^ About 89.1% and 65.2% of patients with 8 W‐RDI ≥50% and ≥ 75% were delivered in this study. On the other hand, atezolizumab 1,200 mg and bevacizumab 500 mg were prescribed intravenously every 3 weeks in patients with A + B. Biochemistry test, including liver function, renal function, serum alpha‐fetoprotein, were checked before the initiation and every 2–4 weeks post therapy. The treatment response was evaluated by dynamic computed tomography (CT) or magnetic resonance imaging (MRI) every 9–12 weeks from initiation of treatment. Radiological responses including initial and best response during therapy were evaluated and classified into complete response (CR), partial response (PR), stable disease (SD), or progressive disease (PD) by radiologist according to Modified Response Evaluation Criteria in Solid Tumors (mRECIST).^14^ All adverse events (AEs) were evaluated according to the Common Terminology Criteria for Adverse Events v5.0 (NCI CTCAE; version 5.0).

### 2.3 Definitions and statistical analysis

Progression free survival (PFS) was defined from the first day of treatment administration to radiological disease progression or death. Overall survival (OS) was calculated from the initial stage of treatment until the date of death. Patients were censored at the date of the last contact or data cutoff date, May 1, 2022, for patients alive without radiological PD. Objective response rate (ORR) and disease control rate (DCR) were defined as CR plus PR and ORR plus SD, respectively.

Descriptive data with normal distribution are presented as mean ± standard deviation; otherwise, they are reported as median (interquartile range, IQR). Independent Student's *t*‐test and Mann–Whitney *U* test were used to assess differences between groups for normal and abnormal distribution variables, sequentially. Statistical analysis was performed using the chi‐square or Fisher's exact test for comparisons of categorical variables between two groups.

In order to adjust the differences of characteristics and to reduce the possible confounding factors between patients receiving lenvatinib and A + B, inverse probability weighting (IPW) analysis were performed. IPW was defined as 1 for the A + B group and (propensity score)/(1−propensity score) for the lenvatinib group adjusting by age, Albumin‐bilirubin index (ALBI), beyond up‐to‐seven (equal or more than seven in the sum of the size of the largest tumor and the number of tumors),^15^ etiology of liver disease, and performance status, respectively. PFS and OS were estimated using the Kaplan–Meier method and compared subgroup using log‐rank test. Predictive factors of PFS, OS, and ORR were determined using a Cox‐regression and logistic regression model, respectively. Two‐tailed P value less than 0.05 was considered statistically significant. Statistical analyses were performed using the SAS version 9.4 and STATA statistical software program (STATA version 14.0; StataCorp).

## 3 RESULTS

### 3.1 Clinical characteristics of enrolled patients

A total of 92 patients including 46 (50%) with lenvatinib and 46 (50%) with A + B were enrolled for analysis. Baseline characteristics of both groups are presented in Table 1. The lenvatinib group had significantly higher proportion of older patients (age **≧**65‐year‐old vs. <65‐year‐old: 65.2 vs. 32.6%), prior locoregional therapy (78.3 vs. 54.4%), lower proportion of beyond up‐to seven (65.4 vs. 89.1%), and higher proportion of AFP **≧** 400 ng/mL (23.9 vs. 62.2%) compared with the A + B group. For reducing the selection biases and confounding variables, IPW analysis was also applied.

**TABLE 1:** Baseline characteristics of the patients

Variables	Overall	Lenvatinib	A + B	*p*‐Value
	No. of patients (*N* = 92)	No. of patients (*N* = 46)	No. of patients (*N* = 46)	
Age	63.8 (38.4–86.9)	69.6 (39.8–86.9)	61.2 (38.4–83.9)	0.0379
≧65	45 (48.9)	30 (65.2)	15 (32.6)	0.0018
Male gender	72 (78.3)	38 (73.1)	38 (82.6)	0.3120
Etiology				
Virus	79 (85.9)	38 (82.6)	41 (89.1)	0.3692
Non‐virus	13 (14.1)	8 (17.4)	5 (10.9)	
ECOG				
0	42 (45.7)	24 (52.2)	18 (39.1)	0.2092
1 or 2	50 (54.3)	22 (47.8)	28 (60.9)	
Child‐Pugh grade (score)				
A (5:6)	81 (57:24)	41 (29:12)	40 (28:12)	0.7480
B (7:8)	11 (6:5)	5 (4:1)	6 (2:4)	
ALBI^a^	−2.49 (IQR − 2.89 to − 2.12)	−2.49 (IQR: −2.85 to − 2.20)	−2.52 (IQR: −2.97 to − 2.01)	0.8493
Grade I	37 (43.0)	17 (39.5)	20 (46.5)	0.5135
Grade II or III	49 (57.0)	26 (60.5)	23 (53.5)	
Macrovascular involvement or portal vein thrombosis	48 (52.2)	24 (52.2)	24 (52.2)	1.0000
Beyond up‐to‐7 criteria	70 (76.1)	34 (65.4)	41 (89.1)	0.0034
Extra‐hepatic metastasis	32 (34.8)	17 (37.0)	15 (32.7)	0.6817
AFP (ng/ml)^b^				
≥ 400	39 (42.9)	11 (23.9)	28 (62.2)	0.0003
BCLC stage				
B	30 (32.6)	16 (34.8)	14 (30.4)	0.6565
C	62 (67.4)	30 (65.2)	32 (69.6)	
Prior locoregional therapy	61 (66.3)	36 (78.3)	25 (54.4)	0.0153
Combine locoregional therapy	35 (38.0)	21 (45.7)	14 (30.4)	0.1328
Observation period (months)	9.4 (IQR: 5.2–13.9)	10.5 (IQR: 5.9–18.2)	8.2 (IQR: 5.1–12.0)	0.0725
Treatment duration (months)	4.7 (IQR: 2.8–8.5)	5.3 (IQR: 2.9–10.4)	4.4 (IQR: 2.8–6.9)	0.1792
Mortality	33 (35.9)	17 (37.0)	16 (34.8)	0.8279
IPW		0.42 (0.26–0.78)	1	0.0678

Abbreviations: A + B, atezolizumab plus bevacizumab; ALBI, albumin‐bilirubin index; AFP, alpha‐fetoprotein; BCLC, Barcelona clinic liver cancer; ECOG PS, Eastern Cooperative Oncology Group performance status; IPW, inverse probability weighting.

^a^ Six patients missing baseline ALBI data.

^b^ One patient missing baseline AFP data.

### 3.2 Treatment response in the lenvatinib group and atezolizumab plus bevacizumab group

We then compared the tumor responses between these two groups. As shown in Table 2, there was no differences between these two groups in terms of ORR (lenvatinib vs. A + B: 26.1% vs. 41.3%, *p* = 0.1226) and DCR (lenvatinib vs. A + B: 63.0% vs. 66.7%, *p* = 0.8279). The similarities of tumor responses were still validated even after IPW in both ORR (lenvatinib vs. A + B: 26.0 vs. 41.3%, *p* = 0.1576) and DCR (lenvatinib vs. A + B: 58.7 vs. 65.2%, *p* = 0.5528).

**TABLE 2:** Best tumor response

	Overall (*N* = 92)	Lenvatinib (*N* = 46)	A + B (*N* = 46)	*p*‐Value
Best response				
Complete response	1 (1.1)	1 (2.2)	0 (0.0)	
Partial response	30 (32.6)	11 (23.9)	19 (41.3)	
Stable disease	28 (30.4)	17 (39.1)	11 (23.9)	
Progressive disease	33 (35.9)	17 (37.0)	16 (34.8)	
Objective response rate	31 (33.7)	12 (26.1)	19 (41.3)	0.1226
Disease control rate	59 (64.1)	29 (63.0)	30 (65.2)	0.8279

Abbreviation: A + B, atezolizumab plus bevacizumab.

**FIGURE 2:**
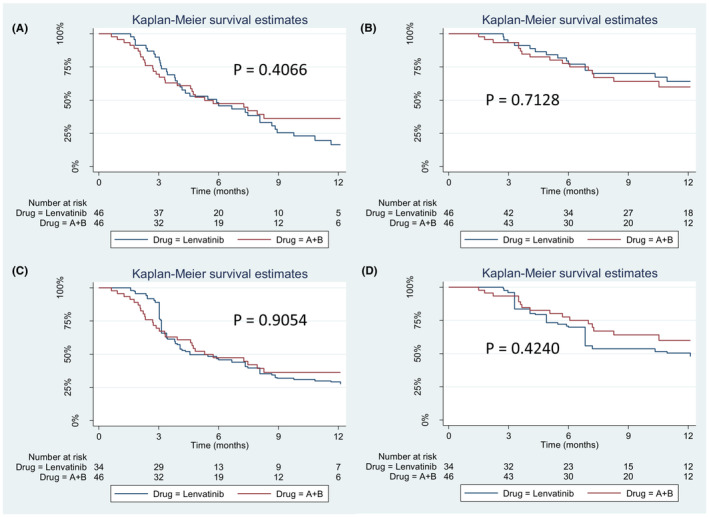
Kaplan–Meier survival function for lenvatinib versus A + B (A) PFS before IPW (B) OS before IPW (C) PFS after IPW (D) OS after IPW. A + B, atezolizumab plus bevacizumab; IPW, inverse probability weighting; OS, overall survival; PFS, progression free survival.

**FIGURE 3:**
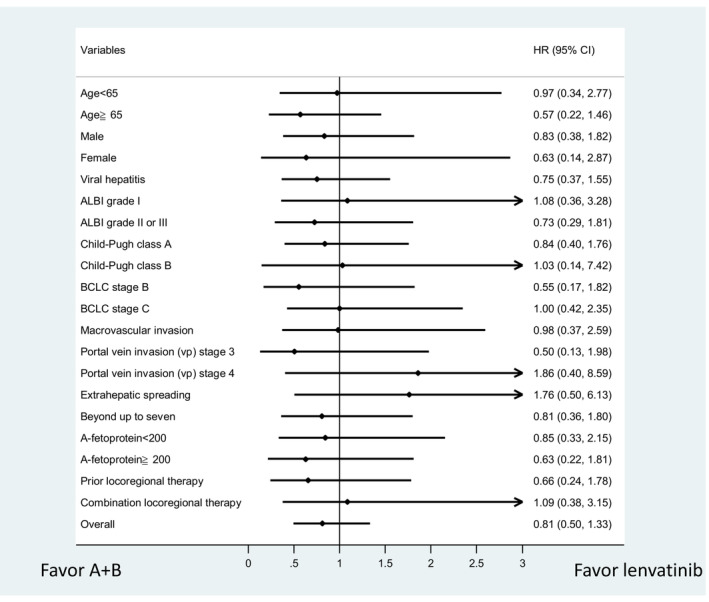
Forrest plot of progression free survival in subgroups analyses. A + B, atezolizumab plus bevacizumab; ALBI, albumin‐bilirubin index; BCLC, Barcelona clinic liver cancer.

In our cohort, the median follow‐up period was 9.4 months with tumor progression observed in 32 patients (69.6%) in the lenvatinib group and 27 patients (58.7%) in the A + B group. The median PFS between lenvatinib and A + B are not significantly different (5.9 vs. 5.3 months, *p* = 0.4066) (Figure 2A). In the post‐IPW cohort, the median PFS was comparable between lenvatinib and A + B (4.6 vs. 5.3 months, *p* = 0.9054) (Figure 2C). The subgroup analysis also show that patients treated with lenvatinib was not inferior to A + B in terms of PFS in all subgroups (Figure 3), including patients with ALBI grade II/III or with high tumor burden such as portal vein invasion (VP3, VP4), or beyond up‐to‐seven. Overall, patients with lenvatinib treatment had propinquity effect on PFS to patients with A + B treatment (HR: 0.812, 95%CI: 0.495–1.331).

For the overall survival, total of 33 mortalities (35.9%) observed during the follow‐up period, including 17 deaths (37.0%) in patients with lenvatinib and 16 deaths (34.8%) in patients with A + B. There was no significant difference of median OS in the lenvatinib and A + B groups whether before IPW (lenvatinib vs. A + B: 22.2 months vs. not reached, p = 0.7128; Figure 2B) or after IPW (lenvatinib vs. A + B: 16.7 months vs. not reached, p = 0.4240; Figure 2D). Subsequent anti‐tumor therapies of lenvatinib and A + B were summarized in Table S1. The median post‐progression survival follow‐up periods were 3.6 and 2.8 months in the lenvatinib and A + B groups, respectively.

### 3.3 Predictors of PFS and OS in the lenvatinib group and atezolizumab plus bevacizumab group

The factors predicting the PFS and OS were analyzed in the overall cohort, lenvatinib, and A + B groups separately. Among the overall patients, there is no independent factor associated with PFS by Cox‐regression model (Table S2). Univariate analysis showed that age and gender were associated with PFS in lenvatinib group whereas ECOG score and macrovascular invasion were predictors in the A + B group. Multivariate analysis showed that age less than 65‐year‐old was an independent predictor and male had a trend associated with longer PFS in the lenvatinib group (adjusted HR = 2.662, 95% CI: 1.157–6.126; *p* = 0.0213; adjusted HR = 0.484, 95% CI: 0.217–1.079; *p* = 0.0762) (Table 3), while ECOG score had a trend association with PFS in the A + B group (adjusted HR = 2.085, 95% CI: 0.914–4.753; *p* = 0.0806) (Table S3).

**TABLE 3:** Predictors for progression free survival in patients received lenvatinib

Variables	All (*N* = 46)	Median PFS (95% CI)	Crude HR (95% CI)	*p* value	Adjust HR (95% CI)	*p* value
Age (years)						
<65	16 (34.8)	8.0667	Referent		Referent	
≧65	30 (65.2)	4.3333	2.918 (1.290–6.601)	0.0101	2.662 (1.157–6.126)	0.0213
Gender						
Female	8 (26.9)	4.0500	Referent		Referent	
Male	38 (73.1)	7.4667	0.409 (0.188–0.890)	0.0242	0.484 (0.217–1.079)	0.0762
Etiology						
Virus	38 (82.6)	6.0000	Referent			
Non‐Virus	8 (17.4)	4.0500	1.350 (0.590–3.090)	0.4775		
ECOG						
0	24 (52.2)	7.3333	Referent			
1 or 2	22 (47.8)	4.1667	1.198 (0.616–2.329)	0.5947		
Child‐Pugh						
A	41 (89.1)	6.0000	Referent			
B	5 (10.9)	3.9333	1.231 (0.431–3.511)	0.6980		
ALBI grade^a^						
I	17 (39.5)	7.3333	Referent			
II or III	26 (60.5)	4.5667	1.226 (0.612–2.455)	0.5658		
Macrovascular involvement or portal vein thrombosis						
No	22 (47.8)	5.7333	Referent			
Yes	24 (52.2)	5.9000	0.931 (0.482–1.799)	0.8325		
Beyond up‐to‐7 criteria						
No	18 (34.6)	8.0667	Referent			
Yes	34 (65.4)	4.3333	1.375 (0.694–2.725)	0.3618		
Extra‐hepatic metastasis						
No	29 (63.0)	4.3333	Referent			
Yes	17 (37.0)	6.6667	0.654 (0.321–1.333)	0.2423		
AFP (ng/ml)						
< 400	35 (76.1)	7.3333	Referent			
≥ 400	11 (23.9)	3.4000	1.834 (0.846–3.977)	0.1247		
BCLC stage						
B	16 (34.8)	3.8333	Referent			
C	30 (65.2)	6.6667	0.707 (0.360–1.391)	0.3157		
Prior locoregional therapy						
No	10 (21.7)	4.5667	Referent			
Yes	36 (78.3)	5.9000	0.976 (0.422–2.256)	0.9544		
Combination locoregional therapy						
No	25 (54.3)	5.9000	Referent			
Yes	21 (45.7)	7.3333	0.865 (0.448–1.673)	0.6668		
Relative dose intensity						
<75% in 8 weeks	16 (34.8)	5.9000	Referent			
≥75% in 8 weeks	30 (65.2)	5.2833	1.356 (0.649–2.832)	0.4173		

Abbreviations: AFP, alpha‐fetoprotein; ALBI, albumin‐bilirubin index; BCLC, Barcelona clinic liver cancer; ECOG PS, Eastern Cooperative Oncology Group performance status.

^a^ Three patients missing baseline ALBI data.

Regarding to OS, ECOG score, Child‐Pugh classification and ALBI score were predictors in univariate analysis whereas multivariate analysis showed ECOG score and Child‐Pugh classification were independent factor in overall patients (ECOG, adjusted HR = 3.348, 95% CI: 1.383–8.106; *p* = 0.0074; Child‐Pugh, adjusted HR = 2.967 95% CI: 1.201–7.332; *p* = 0.0185, respectively) (Table S4). As for the lenvatinib group, not only univariate but also multivariate analysis showed gender, ECOG score, Child‐Pugh classification were predictors (Gender, adjusted HR = 0.288, 95% CI: 0.084–0.989; *p* = 0.0481; ECOG, adjusted HR = 18.284, 95% CI: 1.702–196.418; *p* = 0.0164; Child‐Pugh, adjusted HR = 15.248, 95% CI: 2.534–91.757; *p* = 0.0029, respectively) (Table S5). Among A + B treated patients with ECOG score 0 had a trend of longer OS in univariate analysis (crude HR = 3.366, 95% CI: 0.958–11.826; *p* = 0.0583) (Table S6).

### 3.4 Treatment safety in the lenvatinib group and atezolizumab plus bevacizumab group

**FIGURE 4:**
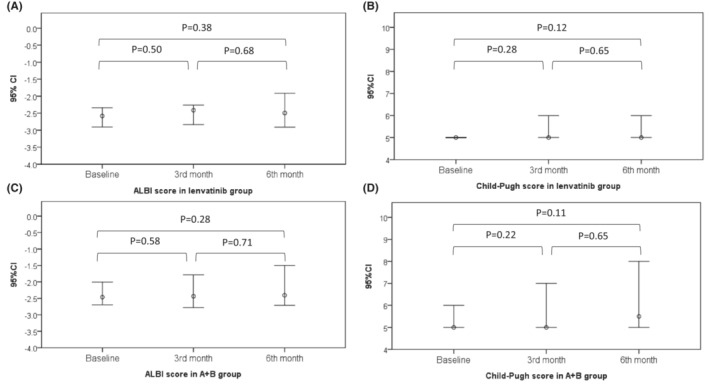
The post‐treatment liver function evaluation in patients with stable disease. (A) ALBI score in lenvatinib group (B) Child‐Pugh score in lenvatinib group (C) ALBI score in atezolizumab plus bevacizumab group (D) Child‐Pugh score in atezolizumab plus bevacizumab. ALBI, albumin‐bilirubin index; A + B, atezolizumab plus bevacizumab.

The incidence rate of any grade treatment‐related adverse event (TRAE) in the lenvatinib group was comparable to the A + B group (76 vs. 63%, *p* = 0.17) (Table 4). Furthermore, lenvatinib treated patients had a trend of higher proportions of severe TRAE (≥ grade 3) (23.9 vs. 10.9%, *p* = 0.0989). In the lenvatinib group, the most frequent TRAE was fatigue, followed by hypertension, hand foot skin rash, anorexia or nausea, proteinuria, and dermatitis. Eleven patients (24%) had occurred severe TRAE, and three of them were hypertension and three were proteinuria. Regarding to the A + B group, the most frequent TRAE were increased aspartate aminotransferase (AST), followed by increased alanine aminotransferase (ALT), fatigue, hypertension, and anorexia or nausea. Among the five patients (11%) with severe TRAE, three of them were with gastrointestinal bleeding including one patient with death of gastrointestinal perforation. The changes of liver reserve in those patients without disease progression were presented in Figure 4. Overall, no statistically significant change of ALBI score and Child‐Pugh score in both the lenvatinib group and A + B group during and after the treatment period.

**TABLE 4:** Treatment‐related adverse events of the overall patients

Adverse events	All (N = 92)		Lenvatinib (N = 46)		A + B (N = 46)	
	Any grade	≥Grade 3	Any grade	≥Grade 3	Any grade	≥Grade 3
Any adverse events	64 (70)	15 (16)	35 (76)	11 (24)	29 (63)	5 (11)
Fatigue	19 (21)	0	13 (28)	0	6 (13)	0
Hypertension	17 (18)	3 (3)	10 (22)	3 (7)	6 (13)	1 (2)
Aspartate aminotransferase increased	15 (16)	0	3 (7)	0	12 (26)	0
Anorexia or nausea	13 (14)	0	8 (17)	0	5 (11)	0
Alanine aminotransferase increased	11 (12)	0	1 (2)	0	10 (22)	1 (2)
Proteinuria	9 (10)	3 (3)	6 (13)	3 (7)	3 (7)	0
Hand foot skin rash	8 (9)	2 (2)	8 (17)	2 (4)	0	0
Dermatitis	8 (9)	1 (1)	6 (13)	1 (2)	2 (4)	0
Dysphonia	5 (5)	0	4 (9)	0	1 (2)	0
Diarrhea	5 (6)	0	3 (7)	0	2 (4)	0
Hyperthyroidism or hypothyroidism	4 (4)	0	2 (4)	0	2 (4)	0
Gastrointestinal bleeding	3 (3)	3 (3)	1 (2)	1 (2)	3 (7)	3 (7)
Abdominal pain	3 (3)	0	3 (7)	0	0	0
Epistaxis or gingiva bleeding	3 (3)	0	0	0	3 (7)	0
Diarrhea	3 (3)	0	0	0	3 (7)	0
Pyrexia	3 (3)	0	0	0	3 (7)	0
Musculoskeletal pain	2 (2)	0	2 (4)	0	0	0
Dizziness or headache	2 (2)	0	1 (2)	0	1 (2)	0
Thrombocytopenia	2 (2)	0	0	0	2 (4)	0
Leukopenia/Neutropenia	2 (2)	0	0	0	2 (4)	0
Blood bilirubin increased	2 (2)	0	0	0	2 (4)	0
Sepsis	1 (1)	1 (1)	1 (2)	1 (2)	0	0
Gastrointestinal perforation	1 (1)	1 (1)	0	0	1 (2)	1 (2)
Alkaline phosphatase increased	1 (1)	0	0	0	1 (2)	0
Infusion reaction	1 (1)	0	0	0	1 (2)	0
Insomnia	1 (1)	0	1 (2)	0	0	0

Abbreviation: A + B, atezolizumab plus bevacizumab.

## 4 DISCUSSION

The first‐line treatment for advanced HCC has been suggested to be A + B as first‐choice, and lenvatinib or sorafenib were alternative choice.^11^ However, the efficacy and side effect between A + B and lenvatinib is still controversial, and more head‐to‐head comparison is still demanded. In our real‐world study, we demonstrated the comparable safety and survival efficacy including PFS and OS between HCC patients receiving the lenvatinib and A + B whether before and after analysis of IPW. These results gave strong evidence to clinician to choose either of these two drugs as the first‐line treatment regimen for the patients with advanced hepatocellular carcinoma.

Recently, there were three retrospective real‐world studies^16^, ^17^, ^18^ and two indirect comparison studies^19^, ^20^ published to investigate the efficacy and safety between lenvatinib and A + B for advanced hepatoma as the first line treatment. However, the results were controversial. As for the ORR, all four real‐world studies including ours showed no significant differences between these two regimens. As for the OS, IMbrave150 showed the superiority of A + B over sorafenib.^7^ In addition, the OS was no difference between lenvatinib and sorafenib in REFLECT trial and subsequent real‐world studies.^3^, ^21^, ^22^ Therefore, it would be reasonable to deduce that the A + B would be better than the lenvatinib in terms of OS. Nevertheless, the results were different in current four studies. Similar OS between two regimens was found in South Korea cohort, one of the propensity score matching‐adjusted Japanese cohort^16^, ^17^ and ours. However, another IPW‐adjusted Japanese cohort showed superior OS rates of A + B was to lenvatinib.^18^ As for the PFS, our study showed equal therapeutic effect between these two regimens with PFS of the lenvatinib group (5.9 months) and A + B group (5.3 months). The results bear a close resemblance to South Korea study which presented 6.0 months of lenvatinib and 5.7 months of A + B and no difference between these two regimens.^16^ The equivalent results may also indirectly be inferenced by PFS of 7.4 months in the lenvatinib group in REFLECT trial and 6.8 months of A + B in IMbrave150 study.^3^, ^7^ On the contrary, both of the Japanese studies showed superior PFS of 8.8 months of A + B compared with 5.2 months of lenvatinib and 0.5−/1−/1.5‐years PFS rates (56.6%/31.6%/non‐estimable) of A + B compared with lenvatinib (48.6%/20.4%/11.2%).^17^, ^18^

Several factors may explain the controversies of OS and PFS in these studies. First, these different results among these studies might be due to differences of patient recruitments. Comparing baseline characteristics of the enrolled patients in these four real‐world studies, it was shown that less proportion of BCLC stage C (50%) and macrovascular invasion (20%) in Japan study.^18^ This difference suggested lower tumor burden in Japan studies and might be the reason for the better outcome in the A + B group. Second, HBV infection was the main etiology of underlying liver disease in South Korea and our study instead of higher proportions of HCV and non‐viral underlying liver disease in Japan studies. Additionally, the different strategies of locoregional therapy may change the anti‐tumor ability. A relative higher proportion of combination with locoregional therapies in than lenvatinib group than in the A + B group although not significant statistically (45.7% vs. 30.4%, *p* = 0.1328) were observed in our study. Recently, TACTICS‐L and LAUNCH trials showed the significant positive result of combination of MKI and TACE.^23^, ^24^ The efficacy and safety of combination with A + B and TACE are investigated in an ongoing clinical trial.^25^ Therefore, a relatively higher proportion of locoregional therapy in the lenvatinib group might improve the OS. Last but not least, post‐progression therapies also play a pivotal role in survival outcome. In our study, there were 37% and 34% patients receiving sequential therapy in the lenvatinib and A + B groups, respectively. Patients in the lenvatinib group received ICI such as nivolumab, pembrolizumab, A + B, or chemotherapy as sequential systemic therapy, whereas MKI such as sorafenib and lenvatinib were the main therapies in the A + B group. Recently, in LEAP‐002 study, the similarity of OS between lenvatinib treatment and lenvatinib plus pembrolizumab treatment was supposed possibly due to a higher proportion of immunotherapy in post‐progression therapy in the lenvatinib group.^26^ However, a further larger prospective design study with longer follow‐up duration should be investigated for confirming these findings.

Although lenvatinib and A + B had been proven their role in HCC treatment, some patients still had poorer liver function reserve who did not meet the trials inclusion criteria. Previous studies demonstrated that lenvatinib and A + B could still be useful for these patients in real‐world practice.^27^, ^28^, ^29^ In our study, there was no difference in terms of PFS between the different regimens in patients with poorer liver function reserve as shown in subgroup analysis by ALBI grading or Child‐Pugh classification. It was also interesting to analyze the differences between different regimens in patients with portal vein invasion VP4 or beyond up‐to‐seven which regard as high tumor burden with relative high‐risk. Sub‐analysis of IMbrave150 trial which enrolled high‐risk groups, both the VP4 and non‐VP4 groups showed equivalent effects on PFS.^30^ Lenvatinib could also offer a survival benefit in VP4 or tumor with more than 50% liver occupation HCC in a real‐world study, although REFLECT trial excluded them as initial study design.^27^ Our subgroup analysis demonstrated that lenvatinib and A + B contributed equivalent treatment efficacy. Therefore, these two regimens could be appropriate treatment choices for high‐risk patients. Regard to etiology of underlying liver disease, lenvatinib had a trend of favorable outcome in HBV infected patients compared with sorafenib, and A + B also showed a better efficacy in patients with viral etiology compared to nonalcoholic steatohepatitis (NASH)‐related HCC.^31^, ^32^ In our study, lenvatinib and A + B showed comparable PFS in viral infected patients. However, the subgroup of NASH‐related HCC analysis cannot be validated due to our small sample size study.

With regard to safety of treatment, we found that lenvatinib had similar occurrence rate of any grade TRAE but higher rate of severe TRAE compared with A + B. Hypertension, proteinuria, and hand foot skin rash are the most reasons of severe TARE in lenvatinib as the same as REFLECT results.^3^ Interestingly, AST and ALT elevations were shown to be more frequent in A + B groups. Immune‐related hepatitis is well recognized and the incidence of liver toxicity is higher in patients who receive combination therapy than in those under monotherapy, although these elevations rarely require patients to stop therapy.^33^ As previous reports, bleeding is also a noteworthy complication due to anti‐VEGF effect in treatment regimens.^7^, ^34^, ^35^ Two patients with A + B had variceal bleeding received endoscopic treatment in the hospital. Hence, pre‐treatment endoscopy to evaluation the risk of varices is recommended.^36^ Three patients with epistaxis or gingiva bleeding and one patient died of gastrointestinal perforation in A + B‐treated patients. Antiangiogenic therapy‐related non‐variceal bleeding should also be a substantial concern. Reserved liver function plays a key role in whether sequential therapy is available or not. Among the patients without disease progression, no significant change of Child‐Pugh score and ALBI score compared with before and third month, sixth month posttreatment evaluation indicated the less influence to liver function in both groups. The results were different from Japan real‐word study which showed significant change of ALBI score at 3 and 6 weeks compared with baseline in the A + B group and increasing ALBI score from baseline, 4 and 8 weeks in the lenvatinib group.^17^ However, in Japanese study, they evaluate the liver functional reserve in overall cohort. In our study, we just studied the patients with stable disease. It is because with tumor progression, liver functions deterioration may occur. Only patients without disease progression were analyzed that accurate reflection of the impact of hepatic reserve by different therapeutic regimens. Furthermore, the increasing risk of HBV reactivation has been reported in HBV infected patients undergoing immunotherapy.^37^ As HBV is the main etiology of underlying liver disease in our cohort, we also found there was no hepatitis B flare or HBV‐related fatal event in this cohort patients. Overall, either lenvatinib or A + B were quite safe without unexpected adverse events.

There are several limitations in our study. First, this is a retrospective study that patients receiving lenvatinib and A + B were not enrolled from the same time. Actually, the baseline characteristics were not significant different before and after year 2020 among the lenvatinib group after cautiously comparing the factors including age, gender, etiology, performance status, liver function, and tumor stage. For minimized selection bias, we tried to use the IPW analysis to adjust the differences of baseline characteristics between the lenvatinib and A + B groups. Second, the sample size in this study was relatively small, which is difficult for further stratification analysis. Current treatment recommendation suggests A + B is the first‐line therapy for advanced HCC.^38^ However, not everyone could afford the A + B due to no reimbursement in Taiwan. Therefore, most of our patients chose lenvatinib treatment. With financial and efficacy evidence reasons, physicians are inclined to choose A + B for patients with higher tumor burden or younger patients. In addition, most patients (85.9%) had chronic viral hepatitis infection and our findings warrant external validation by other cohorts of different HCC etiology or patients' ethnicities. In conclusion, our study suggests that lenvatinib provided a comparable efficacy and safety with A + B for advanced HCC as first‐line therapy. Lenvatinib could serve as an alternative first‐line regimen to A + B especially for its financial benefit.

## AUTHOR CONTRIBUTIONS

**Chung‐Wei Su:** Data curation (equal); formal analysis (equal); investigation (equal); methodology (equal); resources (equal); software (equal); validation (equal); writing – original draft (lead); writing – review and editing (lead). **Wei Teng:** Conceptualization (equal); data curation (supporting); formal analysis (equal); investigation (equal); resources (equal); writing – original draft (equal); writing – review and editing (equal). **Po‐Ting Lin:** Conceptualization (supporting); data curation (supporting); formal analysis (equal); investigation (equal); resources (equal); writing – original draft (supporting); writing – review and editing (supporting). **Wen‐Juei Jeng:** Methodology (equal); supervision (equal); writing – original draft (supporting); writing – review and editing (supporting). **Kuei‐An Chen:** Data curation (equal); validation (equal); writing – original draft (supporting); writing – review and editing (supporting). **Yi‐Chung Hsieh:** Conceptualization (equal); resources (supporting); supervision (equal); writing – original draft (supporting); writing – review and editing (supporting). **Wei Ting Chen:** Resources (equal); supervision (equal); writing – original draft (supporting); writing – review and editing (supporting). **Ming‐Mo Ho:** Investigation (equal); resources (equal); supervision (equal); writing – original draft (supporting); writing – review and editing (supporting). **Jason Chia‐Hsun Hsieh:** Resources (equal); supervision (equal); writing – original draft (supporting); writing – review and editing (supporting). **Ching‐Ting Wang:** Data curation (equal); writing – original draft (supporting). **Pei‐Mei Chai:** Data curation (equal); writing – original draft (supporting). **Chen‐Chun Lin:** Conceptualization (equal); formal analysis (equal); investigation (equal); resources (equal); supervision (equal); writing – original draft (supporting); writing – review and editing (supporting). **Chun‐Yen Lin:** Conceptualization (equal); formal analysis (equal); investigation (equal); supervision (equal); validation (equal); writing – original draft (supporting); writing – review and editing (supporting). **Shi‐Ming Lin:** Conceptualization (equal); investigation (equal); resources (equal); supervision (equal); writing – original draft (supporting); writing – review and editing (supporting).

## FUNDING INFORMATION

None.

## CONFLICT OF INTEREST

The authors have no conflict of interest to declare.

## ETHICAL APPROVAL

Institutional Review Board at our hospital approved this study and the review board waived signed informed consent owing to the retrospective nature of the study.

## Supporting information


Tables S1–S6

## Data Availability

The data that support the findings of this study are available on request from the corresponding author. The data are not publicly available due to privacy or ethical restrictions.
