# Supplementary material for: Similar efficacy and safety between lenvatinib versus atezolizumab plus bevacizumab as the first‐line treatment for unresectable hepatocellular carcinoma
Source: Cancer Med. 2022 Dec 5;12(6):7077–89. doi: 10.1002/cam4.5506 (PMC10067067; doi:10.1002/cam4.5506)
Supplement: Supplementary file 1 — Tables S1–S6 [file CAM4-12-7077-s001.docx]

**Supplementary table A1.** **Subsequent anti-tumor therapy after lenvatinib or A+B**

| **Treatments** | **Lenvatinib**  **No. (%)** | **A+B**  **No. (%)** |
| --- | --- | --- |
| **≥ 1 Anticancer treatment during survival follow-up** | **17(37)** | **16(34)** |
| **Systemic treatment** | **12(26)** | **15(33)** |
| **Sorafenib** | **1(2)** | **5(11)** |
| **Lenvatinib** | **N/A** | **9(20)** |
| **Regorafenib** | **0(0)** | **1(2)** |
| **Ramucirumab** | **0(0)** | **1(2)** |
| **Cabozantinib** | **1(2)** | **0(0)** |
| **A+B** | **2(4)** | **N/A** |
| **Pembrolizumab** | **2(4)** | **2(4)** |
| **Nivolizumab** | **4(9)** | **1(2)** |
| **Chemotherapy** | **3(7)** | **1(2)** |
| **Clinical trials** | **1(2)** | **1(2)** |
| **Locoregional treatment** | **5(11)** | **9(20)** |
| **Transarterial chemoembolization** | **3(7)** | **8(17)** |
| **Radiofrequency Ablation** | **4(9)** | **1(2)** |
| **Hepatic arterial infusion chemotherapy** | **0(0)** | **1(2)** |
| **Surgical resection** | **0(0)** | **1(2)** |

Abbreviations: A+B, atezolizumab plus bevacizumab; N/A, not applicable

**Supplementary table A2. Predictors for progression free survival of the overall patients**

| **Variables** | **All (N=92)** | **Median PFS (95% CI)** | **Crude HR (95% CI)** | **p value** |
| --- | --- | --- | --- | --- |
| **Age (years)** |  |  |  |  |
| **<65** | **47 (51.1)** | **6.6667** | **Referent** |  |
| **≧65** | **45 (48.9)** | **4.7333** | **1.438 (0.870-2.375)** | **0.1566** |
| **Gender** |  |  |  |  |
| **Female** | **20 (21.7)** | **4.7000** | **Referent** |  |
| **Male** | **72 (78.3)** | **7.2667** | **0.817 (0.456-1.464)** | **0.4968** |
| **Etiology** |  |  |  |  |
| **Virus** | **79 (85.9)** | **5.9000** | **Referent** |  |
| **Non-Virus** | **13 (14.1)** | **3.9333** | **1.416 (0.739-2.716)** | **0.2948** |
| **ECOG** |  |  |  |  |
| **0** | **42 (45.7)** | **7.4667** | **Referent** |  |
| **1 or 2** | **50 (54.3)** | **4.1667** | **1.464 (0.891-2.406)** | **0.1323** |
| **Child-Pugh** |  |  |  |  |
| **A** | **81 (88.0)** | **6.0000** | **Referent** |  |
| **B** | **11 (12.0)** | **3.4000** | **1.278 (0.607-2.691)** | **0.5191** |
| **ALBI grade†** |  |  |  |  |
| **I** | **37 (43.0)** | **5.9000** | **Referent** |  |
| **II or III** | **49 (57.0)** | **5.3000** | **1.175 (0.703-1.964)** | **0.5388** |
| **Macrovascular involvement or portal vein thrombosis** |  |  |  |  |
| **No** | **48 (52.2)** | **7.2667** | **Referent** |  |
| **Yes** | **44 (47.8)** | **4.7333** | **1.345 (0.819-2.208)** | **0.2419** |
| **Beyond up-to-7 criteria** |  |  |  |  |
| **No** | **22 (23.9)** | **8.0667** | **Referent** |  |
| **Yes** | **70 (76.1)** | **4.8333** | **1.256 (0.711-2.220)** | **0.4326** |
| **Extra-hepatic metastasis** |  |  |  |  |
| **No** | **60 (65.2)** | **5.7333** | **Referent** |  |
| **Yes** | **32 (34.8)** | **5.4667** | **0.854 (0.506-1.443)** | **0.5561** |
| **AFP (ng/ml)** **‡** |  |  |  |  |
| **<400** | **52 (57.1)** | **7.3333** | **Referent** |  |
| **≥400** | **39 (42.9)** | **4.6667** | **1.300 (0.791-2.136)** | **0.3002** |
| **BCLC stage** |  |  |  |  |
| **B** | **30 (32.6)** | **4.3333** | **Referent** |  |
| **C** | **62 (67.4)** | **5.7333** | **1.004 (0.595-1.693)** | **0.9884** |
| **Prior locoregional therapy** |  |  |  |  |
| **No** | **31 (23.7)** | **4.6667** | **Referent** |  |
| **Yes** | **61 (66.3)** | **7.4667** | **0.824 (0.491-1.382)** | **0.4624** |
| **Combination locoregional therapy** |  |  |  |  |
| **No** | **57 (62.0)** | **6.0000** | **Referent** |  |
| **Yes** | **35 (38.0)** | **4.8333** | **1.214 (0.739-1.997)** | **0.4439** |
| **Treatment** |  |  |  |  |
| **Lenvatinib** | **46 (50.0)** | **5.9000** | **Referent** |  |
| **A+B** | **46 (50.0)** | **5.3000** | **0.812 (0.495-1.331)** | **0.4083** |

Abbreviations: ECOG PS, Eastern Cooperative Oncology Group performance status; ALBI, Albumin-bilirubin index; AFP, alpha-fetoprotein; BCLC, Barcelona Clinic Liver Cancer; A+B, atezolizumab plus bevacizumab

†Six patients missing baseline ALBI data ‡One patient missing baseline AFP data

**Supplementary table A3. Predictors for progression free survival in patients with atezolizumab plus bevacizumab**

| **Variables** | **All (N=46)** | **Median PFS (95% CI)** | **Crude HR (95% CI)** | **p value** | **Adjust HR (95% CI)** | **p value** |
| --- | --- | --- | --- | --- | --- | --- |
| **Age (years)** |  |  |  |  |  |  |
| **<65** | **31 (67.4)** | **5.3000** | **Referent** |  |  |  |
| **≧65** | **15 (32.6)** | **7.9333** | **0.746 (0.328-1.695)** | **0.4836** |  |  |
| **Gender** |  |  |  |  |  |  |
| **Female** | **8 (17.4)** | **NR** | **Referent** |  |  |  |
| **Male** | **38 (82.6)** | **5.3000** | **1.517 (0.525-4.380)** | **0.4413** |  |  |
| **Etiology** |  |  |  |  |  |  |
| **Virus** | **41 (89.1)** | **5.7333** | **Referent** |  |  |  |
| **Non-Virus** | **5 (10.9)** | **3.9333** | **1.606 (0.556-4.642)** | **0.3816** |  |  |
| **ECOG** |  |  |  |  |  |  |
| **0** | **18 (39.1)** | **NR** | **Referent** |  | **Referent** |  |
| **1 or 2** | **28 (60.9)** | **4.2667** | **2.157 (0.948-4.907)** | **0.0670** | **2.085 (0.914-4.753)** | **0.0806** |
| **Child-Pugh** |  |  |  |  |  |  |
| **A** | **40 (87.0)** | **5.7333** | **Referent** |  |  |  |
| **B** | **6 (13.0)** | **3.0000** | **1.413 (0.488-4.090)** | **0.5236** |  |  |
| **ALBI grade†** |  |  |  |  |  |  |
| **I** | **20 (46.5)** | **5.7333** | **Referent** |  |  |  |
| **II or III** | **23 (53.5)** | **5.3000** | **1.140 (0.525-2.472)** | **0.7407** |  |  |
| **Macrovascular involvement or portal vein thrombosis** |  |  |  |  |  |  |
| **No** | **22 (47.8)** | **8.2667** | **Referent** |  | **Referent** |  |
| **Yes** | **24 (52.2)** | **4.7000** | **1.930 (0.895-4.160)** | **0.0934** | **1.851 (0.861-3.980)** | **0.1151** |
| **Beyond up-to-7 criteria** |  |  |  |  |  |  |
| **No** | **5 (12.2)** | **NR** | **Referent** |  |  |  |
| **Yes** | **41 (89.1)** | **5.3000** | **1.954 (0.463-8.243)** | **0.3616** |  |  |
| **Extra-hepatic metastasis** |  |  |  |  |  |  |
| **No** | **31 (67.4)** | **7.2667** | **Referent** |  |  |  |
| **Yes** | **15 (32.6)** | **4.8333** | **1.210 (0.558-2.624)** | **0.6287** |  |  |
| **AFP (ng/ml) ‡** |  |  |  |  |  |  |
| **<400** | **17 (37.8)** | **7.9333** | **Referent** |  |  |  |
| **≥400** | **28 (62.2)** | **5.0667** | **1.335 (0.603-2.953)** | **0.4758** |  |  |
| **BCLC stage** |  |  |  |  |  |  |
| **B** | **14 (30.4)** | **7.9333** | **Referent** |  |  |  |
| **C** | **32 (69.6)** | **4.7833** | **1.603 (0.681-3.774)** | **0.2800** |  |  |
| **Prior locoregional therapy** |  |  |  |  |  |  |
| **No** | **21 (45.6)** | **4.6667** | **Referent** |  |  |  |
| **Yes** | **25 (54.4)** | **7.9333** | **0.582 (0.277-1.227)** | **0.1549** |  |  |
| **Combination locoregional therapy** |  |  |  |  |  |  |
| **No** | **32 (69.6)** | **7.2667** | **Referent** |  |  |  |
| **Yes** | **14 (30.4)** | **4.7833** | **1.568 (0.731-3.363)** | **0.2481** |  |  |

Abbreviations: ECOG PS, Eastern Cooperative Oncology Group performance status; ALBI, Albumin-bilirubin index; AFP, alpha-fetoprotein; BCLC, Barcelona Clinic Liver Cancer

†Three patients missing baseline ALBI data ‡One patient missing baseline AFP data

**Supplementary table A4. Predictors for overall survival in overall patients**

| **Variables** | **All (N=92)** | **Median OS (95% CI)** | **Crude HR (95% CI)** | **p value** | **Adjust HR (95% CI)** | **p value** |
| --- | --- | --- | --- | --- | --- | --- |
| **Age (years)** |  |  |  |  |  |  |
| **<65** | **47 (51.1)** | **22.3** | **Referent** |  |  |  |
| **≧65** | **45 (48.9)** | **NR** | **0.888 (0.447-1.767)** | **0.7361** |  |  |
| **Gender** |  |  |  |  |  |  |
| **Female** | **20 (21.7)** | **10.3** | **Referent** |  | **Referent** |  |
| **Male** | **72 (78.3)** | **NR** | **0.494 (0.233-1.046)** | **0.0654** | **0.634 (0.288-1.397)** | **0.2587** |
| **Etiology** |  |  |  |  |  |  |
| **Virus** | **79 (85.9)** | **NR** | **Referent** |  |  |  |
| **Non-Virus** | **13 (14.1)** | **16.7000** | **1.622 (0.726-3.623)** | **0.2384** |  |  |
| **ECOG** |  |  |  |  |  |  |
| **0** | **42 (45.7)** | **NR** | **Referent** |  | **Referent** |  |
| **1 or 2** | **50 (54.3)** | **10.5333** | **3.853 (1.719-8.636)** | **0.0011** | **3.348 (1.383-8.106)** | **0.0074** |
| **Child-Pugh** |  |  |  |  |  |  |
| **A** | **81 (88.0)** | **NR** | **Referent** |  | **Referent** |  |
| **B** | **11 (12.0)** | **4.3333** | **3.315 (1.482-7.415)** | **0.0035** | **2.967 (1.201-7.332)** | **0.0185** |
| **ALBI grade†** |  |  |  |  |  |  |
| **I** | **37 (43.0)** | **NR** | **Referent** |  | **Referent** |  |
| **II or III** | **49 (57.0)** | **10.9333** | **2.680 (1.183-6.068)** | **0.0181** | **1.539 (0.628-3.775)** | **0.3460** |
| **Macrovascular involvement or portal vein thrombosis** |  |  |  |  |  |  |
| **No** | **48 (52.2)** | **NR** | **Referent** |  |  |  |
| **Yes** | **44 (47.8)** | **NR** | **1.310 (0.652-2.632)** | **0.4488** |  |  |
| **Beyond up-to-7 criteria** |  |  |  |  |  |  |
| **No** | **22 (23.9)** | **NR** | **Referent** |  |  |  |
| **Yes** | **70 (76.1)** | **NR** | **2.027 (0.810-5.076)** | **0.1313** |  |  |
| **Extra-hepatic metastasis** |  |  |  |  |  |  |
| **No** | **60 (65.2)** | **NR** | **Referent** |  |  |  |
| **Yes** | **32 (34.8)** | **22.2333** | **1.198 (0.595-2.412)** | **0.6124** |  |  |
| **AFP (ng/ml)** **‡** |  |  |  |  |  |  |
| **<400** | **52 (57.1)** | **22.2333** | **Referent** |  |  |  |
| **≥400** | **39 (42.9)** | **NR** | **1.327 (0.665-2.648)** | **0.4215** |  |  |
| **BCLC stage** |  |  |  |  |  |  |
| **B** | **30 (32.6)** | **NR** | **Referent** |  |  |  |
| **C** | **62 (67.4)** | **22.2333** | **1.905 (0.857-4.232)** | **0.1135** |  |  |
| **Prior locoregional therapy** |  |  |  |  |  |  |
| **No** | **31 (23.7)** | **NR** | **Referent** |  | **Referent** |  |
| **Yes** | **61 (66.3)** | **NR** | **0.533 (0.263-1.078)** | **0.0798** | **0.697 (0.329-1.477)** | **0.3456** |
| **Combination locoregional therapy** |  |  |  |  |  |  |
| **No** | **57 (62.0)** | **22.2333** | **Referent** |  |  |  |
| **Yes** | **35 (38.0)** | **NR** | **0.758 (0.367-1.568)** | **0.4556** |  |  |
| **Treatment** |  |  |  |  |  |  |
| **Lenvatinib** | **46 (50.0)** | **22.2333** | **Referent** |  |  |  |
| **A+B** | **46 (50.0)** | **NR** | **1.140 (0.568-2.289)** | **0.7128** |  |  |

Abbreviations: ECOG PS, Eastern Cooperative Oncology Group performance status; ALBI, Albumin-bilirubin index; AFP, alpha-fetoprotein; BCLC, Barcelona Clinic Liver Cancer; A+B, atezolizumab plus bevacizumab

†Six patients missing baseline ALBI data ‡One patient missing baseline AFP data

**Supplementary table A5. Predictors for overall survival in patients received lenvatinib**

| **Variables** | **All (N=46)** | **Median PFS (95% CI)** | **Crude HR (95% CI)** | **p value** | **Adjust HR (95% CI)** | **p value** |
| --- | --- | --- | --- | --- | --- | --- |
| **Age (years)** |  |  |  |  |  |  |
| **<65** | **16 (34.8)** | **22.2333** | **Referent** |  |  |  |
| **≧65** | **30 (65.2)** | **NR** | **1.145 (0.421-3.113)** | **0.7913** |  |  |
| **Gender** |  |  |  |  |  |  |
| **Female** | **8 (26.9)** | **6.8333** | **Referent** |  | **Referent** |  |
| **Male** | **38 (73.1)** | **NR** | **0.214 (0.079-0.583)** | **0.0026** | **0.288 (0.084-0.989)** | **0.0481** |
| **Etiology** |  |  |  |  |  |  |
| **Virus** | **38 (82.6)** | **16.7000** | **Referent** |  |  |  |
| **Non-Virus** | **8 (17.4)** | **NR** | **1.676 (0.584-4.805)** | **0.3369** |  |  |
| **ECOG** |  |  |  |  |  |  |
| **0** | **24 (52.2)** | **NR** | **Referent** |  | **Referent** |  |
| **1 or 2** | **22 (47.8)** | **6.8333** | **4.536 (1.544-13.323)** | **0.0058** | **18.284 (1.702-196.418)** | **0.0164** |
| **Child-Pugh** |  |  |  |  |  |  |
| **A** | **41 (89.1)** | **NR** | **Referent** |  | **Referent** |  |
| **B** | **5 (10.9)** | **4.3333** | **4.578 (1.457-14.379)** | **0.0092** | **15.248 (2.534-91.757)** | **0.0029** |
| **ALBI grade†** |  |  |  |  |  |  |
| **I** | **17 (39.5)** | **NR** | **Referent** |  | **Referent** |  |
| **II or III** | **26 (60.5)** | **NR** | **2.818 (0.890-8.926)** | **0.0782** | **0.138 (0.010-1.916)** | **0.1402** |
| **Macrovascular involvement or portal vein thrombosis** |  |  |  |  |  |  |
| **No** | **22 (47.8)** | **NR** | **Referent** |  |  |  |
| **Yes** | **24 (52.2)** | **NR** | **1.183 (0.442-3.165)** | **0.7373** |  |  |
| **Beyond up-to-7 criteria** |  |  |  |  |  |  |
| **No** | **18 (34.6)** | **NR** | **Referent** |  | **Referent** |  |
| **Yes** | **34 (65.4)** | **10.9333** | **3.046 (0.954-9.732)** | **0.0602** | **0.950 (0.194-4.659)** | **0.9496** |
| **Extra-hepatic metastasis** |  |  |  |  |  |  |
| **No** | **29 (63.0)** | **NR** | **Referent** |  |  |  |
| **Yes** | **17 (37.0)** | **22.2333** | **0.914 (0.337-2.478)** | **0.8593** |  |  |
| **AFP (ng/ml)** |  |  |  |  |  |  |
| **<400** | **35 (76.1)** | **NR** | **Referent** |  |  |  |
| **≥400** | **11 (23.9)** | **6.8333** | **1.799 (0.630-5.140)** | **0.2727** |  |  |
| **BCLC stage** |  |  |  |  |  |  |
| **B** | **16 (34.8)** | **NR** | **Referent** |  |  |  |
| **C** | **30 (65.2)** | **22.2333** | **1.569 (0.551-4.468)** | **0.3991** |  |  |
| **Prior locoregional therapy** |  |  |  |  |  |  |
| **No** | **10 (21.7)** | **NR** | **Referent** |  |  |  |
| **Yes** | **36 (78.3)** | **NR** | **0.540 (0.185-1.576)** | **0.2594** |  |  |
| **Combination locoregional therapy** |  |  |  |  |  |  |
| **No** | **25 (54.3)** | **22.2333** | **Referent** |  |  |  |
| **Yes** | **21 (45.7)** | **NR** | **0.815 (0.307-2.164)** | **0.6816** |  |  |
| **Relative dose intensity** |  |  |  |  |  |  |
| **<75% in 8 weeks** | **16 (34.8)** | **NR** | **Referent** |  |  |  |
| **≥75% in 8 weeks** | **30 (65.2)** | **22.2333** | **1.214 (0.424-3.471)** | **0.7180** |  |  |

Abbreviations: ECOG PS, Eastern Cooperative Oncology Group performance status; ALBI, Albumin-bilirubin index; AFP, alpha-fetoprotein; BCLC, Barcelona Clinic Liver Cancer

†Three patients missing baseline ALBI data

**Supplementary table A6. Predictors for overall survival in patients with atezolizumab plus bevacizumab**

| **Variables** | **All (N=46)** | **Median PFS (95% CI)** | **Crude HR (95% CI)** | **p value** | **Adjust HR (95% CI)** | **p value** |
| --- | --- | --- | --- | --- | --- | --- |
| **Age (years)** |  |  |  |  |  |  |
| **<65** | **31 (67.4)** | **NR** | **Referent** |  |  |  |
| **≧65** | **15 (32.6)** | **NR** | **0.686 (0.221-2.128)** | **0.5140** |  |  |
| **Gender** |  |  |  |  |  |  |
| **Female** | **8 (17.4)** | **NR** | **Referent** |  |  |  |
| **Male** | **38 (82.6)** | **NR** | **1.562 (0.355-6.876)** | **0.5556** |  |  |
| **Etiology** |  |  |  |  |  |  |
| **Virus** | **41 (89.1)** | **NR** | **Referent** |  |  |  |
| **Non-Virus** | **5 (10.9)** | **10.5333** | **1.631 (0.461-5.768)** | **0.4476** |  |  |
| **ECOG** |  |  |  |  |  |  |
| **0** | **18 (39.1)** | **NR** | **Referent** |  |  |  |
| **1 or 2** | **28 (60.9)** | **10.5333** | **3.366 (0.958-11.826)** | **0.0583** |  |  |
| **Child-Pugh** |  |  |  |  |  |  |
| **A** | **40 (87.0)** | **NR** | **Referent** |  |  |  |
| **B** | **6 (13.0)** | **7.3000** | **2.469 (0.794-7.676)** | **0.1183** |  |  |
| **ALBI grade†** |  |  |  |  |  |  |
| **I** | **20 (46.5)** | **NR** |  |  |  |  |
| **II or III** | **23 (53.5)** | **10.5333** | **2.567 (0.803-8.203)** | **0.1118** |  |  |
| **Macrovascular involvement or portal vein thrombosis** |  |  |  |  |  |  |
| **No** | **22 (47.8)** | **NR** | **Referent** |  |  |  |
| **Yes** | **24 (52.2)** | **10.5333** | **1.443 (0.534-3.901)** | **0.4694** |  |  |
| **Beyond up-to-7 criteria** |  |  |  |  |  |  |
| **No** | **5 (12.2)** | **NR** | **Referent** |  |  |  |
| **Yes** | **41 (89.1)** | **NR** | **0.841 (0.191-3.704)** | **0.8188** |  |  |
| **Extra-hepatic metastasis** |  |  |  |  |  |  |
| **No** | **31 (67.4)** | **NR** | **Referent** |  |  |  |
| **Yes** | **15 (32.6)** | **NR** | **1.602 (0.596-4.309)** | **0.3505** |  |  |
| **AFP (ng/ml) ‡** |  |  |  |  |  |  |
| **<400** | **17 (37.8)** | **NR** | **Referent** |  |  |  |
| **≥400** | **28 (62.2)** | **NR** | **0.940 (0.341-2.593)** | **0.9055** |  |  |
| **BCLC stage** |  |  |  |  |  |  |
| **B** | **14 (30.4)** | **NR** | **Referent** |  |  |  |
| **C** | **32 (69.6)** | **NR** | **2.379 (0.677-8.364)** | **0.1766** |  |  |
| **Prior locoregional therapy** |  |  |  |  |  |  |
| **No** | **21 (45.6)** | **NR** | **Referent** |  |  |  |
| **Yes** | **25 (54.4)** | **NR** | **0.517 (0.191-1.396)** | **0.1928** |  |  |
| **Combination locoregional therapy** |  |  |  |  |  |  |
| **No** | **32 (69.6)** | **NR** | **Referent** |  |  |  |
| **Yes** | **14 (30.4)** | **NR** | **0.704 (0.227-2.186)** | **0.5442** |  |  |

Abbreviations: ECOG PS, Eastern Cooperative Oncology Group performance status; ALBI, Albumin-bilirubin index; AFP, alpha-fetoprotein; BCLC, Barcelona Clinic Liver Cancer

†Six patients missing baseline ALBI data ‡One patient missing baseline AFP data
